# Insulin Resistance in Pediatric Obesity: From Mechanisms to Treatment Strategies

**DOI:** 10.1155/2024/2298306

**Published:** 2024-06-28

**Authors:** Yu Luo, Dan Luo, Maojun Li, Binzhi Tang

**Affiliations:** ^1^ Department of Pediatrics Sichuan Provincial People's Hospital School of Medicine University of Electronic Science and Technology of China, Chengdu, China; ^2^ Department of Pediatrics School of Medicine and Life Science of Chengdu University of Traditional Chinese Medicine, Chengdu, China

## Abstract

Insulin resistance, an increasingly prevalent characteristic among children and adolescents with obesity, is now recognized as a significant contributor to the development of type 2 diabetes mellitus (T2DM) and other metabolic diseases in individuals with obesity. Insulin resistance refers to a decrease in the sensitivity of peripheral tissues (primarily skeletal muscle, adipose tissue, and liver) to insulin, which is mainly characterized by impaired glucose uptake and utilization. Although the mechanisms underlying insulin resistance in children with obesity remain incompletely elucidated, several risk factors including lipid metabolism disorders, oxidative stress (OS), mitochondrial dysfunction, inflammation, and genetic factors have been identified as pivotal contributors to the pathogenesis of obesity-related insulin resistance. In this review, we comprehensively analyze relevant literature and studies to elucidate the underlying mechanisms of insulin resistance in childhood obesity. Additionally, we discuss treatment strategies for pediatric obesity from a perspective centered on improving insulin sensitivity, aiming to provide valuable insights for the prevention and management of pediatric obesity.

## 1. Introduction

Childhood and adolescent overweight or obesity has become a global health concern, predominantly concentrated in low-income countries, particularly in the Pacific island regions [1]. Pediatric obesity is considered a crucial risk factor for the development of chronic conditions such as type 2 diabetes mellitus (T2DM), hypertension, hyperlipidemia, fatty liver, and metabolic syndrome (MS) in children and adolescents, which also increases the likelihood of developing chronic diseases in adulthood. It has been found that free fatty acids (FFA), inflammatory factors, mitochondrial dysfunction, endoplasmic reticulum stress (ERS), and other factors act independently or in concert during the development of insulin resistance, leading to obesity and its associated complications [2]. Clarifying these mechanisms is beneficial for the improvement of insulin resistance in childhood and adolescent obesity, thereby reducing the incidence of associated metabolic diseases [3, 4] This review aims to improve the prophylaxis and management of pediatric obesity by focusing on the interplay between childhood obesity and insulin resistance, with a view to elucidate the molecular mechanisms and treatment strategies.

## 2. Pediatric Obesity

Pediatric obesity, a multifactorial disease, is characterized by excessive fat accumulation and results from the interplay of genetic, environmental, and social factors [5]. Currently, ~2.2 billion individuals worldwide are overweight and 712 million are obese, alarmingly, more than 100 million children are suffering from obesity [6]. As a consequence, childhood obesity has been recognized by the World Health Organization (WHO) as one of the foremost challenges in the 21st century. The classification of obesity is commonly based on body mass index (BMI), whereby adults are categorized as overweight if their BMI ranges from 25 to 30, and as obese if it exceeds 30 [7]. However, BMI varies greatly with age throughout childhood and adolescence, rendering the simple calculation of dividing body weight in kilograms by height in meters squared (kg/m^2^) inaccurate for evaluating childhood obesity. BMI exhibits high specificity in detecting obesity, but it demonstrates low sensitivity and fails to accurately characterize the distribution of body fat. The low sensitivity can now be enhanced by calculating BMIz-score (BMIz) and percentiles, however, the inherent limitation in accurately characterizing the distribution of body fat remains insurmountable [8]. The recently published “*Clinical Practice Guideline for the Evaluation and Treatment of Children and Adolescents*” by the American Academy of Pediatrics (AAP) confirms that utilizing sex-specific BMI-for-age growth charts remains the prevailing standard for assessing obesity in children and adolescents in the United States. For children and adolescents ages 2–18 years, those with sex-specific BMI-for-age growth charts between the 85th and 95th percentiles are categorized as overweight, while individuals above the 95th percentile are classified as obese. Moreover, those with sex-specific BMI-for-age growth charts exceeding 120% of the 95th percentile for both age and gender are considered severely obese. The Centers for Disease Control and Prevention (CDC) and the AAP recommend utilizing the WHO's weight-for-length, age-, and sex-specific charts to monitor weight status in children under 2 years old. Specialized growth charts are available for children and adolescents with specific conditions, such as trisomy 21, which can provide valuable growth reference information for special populations [8]. In general, obesity significantly impacts the growth and development of children and adolescents, causing harm to organ systems and psychological intelligence, particularly the cardiovascular and endocrine systems [9].

Childhood obesity is influenced by various factors, encompassing genetics, food, physical exercise, gut microbiota composition, lifestyle, and cognitive development. The heritability of body weight is relatively high, with genetic variations playing a significant role in determining an individual's susceptibility or resistance to obesogenic environments [1]. As the adiposity-related genes are relatively stable, childhood obesity is predominantly influenced by behavioral and environmental factors. In modern society, high-fat diets, excessive sugar intake, sedentary lifestyles, and staying up late are critical contributors to insulin resistance and the exacerbation of obesity in children and adolescents. Therefore, enhancing behavioral interventions and exercise regimens is beneficial for the prevention of childhood obesity.

## 3. Insulin Resistance and T2DM

Insulin is the only hypoglycemic hormone involved in regulating glucose and fat metabolism in the body. Insulin resistance arises when peripheral tissues, such as skeletal muscle, liver, and adipose tissue exhibit decreased sensitivity to insulin, resulting in impaired uptake and utilization of glucose [10]. More than 90% of primary insulin resistance is attributed to multiple genetic mutations such as those affecting the *glucose transporter type 4* (*GLUT4*), *glucokinase*, and *insulin receptor substrate* gene. Conversely, secondary insulin resistance is predominantly influenced by environmental factors, with obesity being recognized as one of the most significant. The estimated likelihood of children with obesity developing insulin resistance is 38.7%, and the accumulation of visceral fat in their bodies independently contributes to insulin sensitivity [11]. Excessive adiposity significantly and persistently affects insulin sensitivity in adipose tissue, skeletal muscle, and liver among children with obesity, and insulin resistance may arise in some of these tissues prior to the onset of T2DM in individuals with obesity [12, 13].

Insulin resistance induced hyperinsulinemia is common in children and adolescents with obesity. The vicious cycle of insulin resistance and chronic exposure to excess glucose and FFA ultimately leads to *β* cell damage, impairing the capacity for insulin synthesis and secretion. This eventually results in *β* cell apoptosis and the onset of T2DM [14]. Indeed, the onset of diabetes is showing a trend toward an earlier age. The global prevalence of T2DM in children and adolescents is positively correlated with the escalating rates of childhood obesity, and the vast majority of children with T2DM are overweight or obese [15]. Moreover, Fang et al. found that the majority of children with obesity and diabetes are at a significantly increased risk of developing diabetes in adulthood [16]. The escalating prevalence of childhood obesity has led to the transformation of T2DM from an exclusive adult disease into a critical pediatric health concern [17]. T2DM is a metabolic disease characteristic of insulin insufficiency, often accompanied by metabolic disorders such as hyperglycemia, insulin resistance, and hyperlipidemia. Unlike type 1 diabetes mellitus (T1DM) in children and adolescents or T2DM in adults, the development of T2DM in children is characterized by a rapid decline in *β* cell function, early onset, and accelerated progression of complications [12]. The prevalence of complications, including nephropathy, hypertension, lipid metabolism disorders, retinopathy, and neuropsychiatric disorders, is significantly higher in underage patients due to their poor glycemic control. Among these complications, renal disease is considered to be the most common and earliest manifestation. Insulin resistance not only contributes to the development of obesity and T2DM but also plays a role in the pathogenesis of hypertension, dyslipidemia, MS, and cardiovascular disease. Therefore, it is crucial for child health practitioners and families to be aware of this association.

### 3.1. Indictors for Predicting Insulin Resistance

Timely evaluation of the insulin secretion function of islets in children with obesity is essential for early intervention to prevent diabetes. The blood insulin-glucose clamp technique has been regarded as the golden standard method for evaluating insulin resistance due to its exceptional accuracy and repeatability; however, its complexity limits its application in measuring large samples. Current instruments employed for assessing insulin resistance in both epidemiological studies and clinical practice include the homeostasis model assessment of insulin resistance (HOMA-IR), HOMA of percentage beta-cell function (HOMA-*β*), and quantitative insulin sensitivity test index (QUICKI) [18]. The triglyceride–glucose index (TyG-index) is commonly employed in large-scale studies or for identifying high-risk populations for diabetes due to its superior sensitivity in predicting insulin resistance, as compared to HOMA-IR [19]. The influence of gender on indicators for predicting insulin resistance, particularly during mid-adolescence, should be acknowledged, as it may be associated with variations in sex hormone levels, development, and body fat distribution [20]. A recent meta-analysis has confirmed that adolescents aged 12–18 years with obesity are more likely to develop insulin resistance, and related indicators such as insulin, C-peptide, and HOMA-IR index were also elevated [21].

Currently, HOMA-IR serves as a reliable indicator of insulin resistance by providing an estimation based on fasting blood glucose and serum insulin concentration. The HOMA-IR index increases proportionally with the rise in insulin resistance. Furthermore, standardizing the measurement of fasting insulin is imperative to mitigate potential biases [19]. The comparative evaluation values of fasting insulin, HOMA-IR, and blood insulin-glucose clamp techniques for assessing insulin resistance have been demonstrated. Pires and colleagues discovered a positive correlation between HOMA-IR and various indicators including BMI, waist–hip ratio, body percentage, body weight, body fat, and visceral fat area. Among them, the waist–hip ratio exhibited the strongest association with HOMA-IR levels, highlighting central obesity as a significant risk factor for insulin resistance [20]. However, there is no definitive cutoff value for HOMA-IR due to geographic and ethnographic differences, a comprehensive study conducted in 2019 involving a large sample of Czech adults identified a specific HOMA-IR cutoff point of 3.63, while Italian children have an established HOMA-IR standard >4 [22].

### 3.2. Adolescence and Insulin Resistance

The period of adolescence is characterized by rapid physical and sexual maturation and an increased prevalence of obesity as well [20]. A transient increase in insulin resistance commonly occurs during puberty, typically manifesting at the onset of puberty and returning to baseline levels at the end of puberty. This perspective has been supported by research conducted on healthy populations which indicates that increased insulin secretion is inversely related to insulin sensitivity, and physiological insulin sensitivity declines during puberty regardless of gender, ethnicity, weight, or sex hormone levels [23]. Insulin resistance is influenced by various factors including BMI and body fat distribution, and primarily driven by the growth hormone/insulin-like growth factor-1 (GH/IGF-1) axis during adolescence. Elevated serum level of GH/IGF-1 during puberty is positively associated with increased insulin resistance [24]. Physiological insulin resistance occurs during early puberty accompanied by hyperandrogenic manifestations resulting from increased GH secretion [25]. IGF-1 levels have been found to be associated with insulin sensitivity in both boys and girls, but only in girls IGF-1 is significantly influenced by body fat and strongly correlated with fasting insulin levels, especially in lean girls. These findings suggested that the GH/IGF-1 axis plays a significant role in the development of insulin resistance during adolescence [18].

Early studies have demonstrated that the occurrence of insulin resistance at age 13 is associated with a higher risk of hypertension and hypertriglyceridemia at age 19, highlighting childhood insulin resistance as an essential predictor of cardiovascular disease for the first time [26]. Recently, researchers have drawn a causal link between adolescent atherosclerosis and early adulthood hyperinsulinemia and insulin resistance based on data from the original Avon Longitudinal Study of Parents and Children (ALSPAC) cohort study [27]. Similarly, follow-up of up to 7 years has shown that atherosclerosis in adolescence may precede low HDL-cholesterol in young adults. Although the statistical significance of these findings was only marginal, they suggest that preventing and treating insulin resistance, early-onset T2DM, and dyslipidemia concurrently may be effective strategies for reducing atherosclerosis, particularly when initiated during adolescence [27, 28]. A 9 year longitudinal prospective cohort study conducted in the United Kingdom has confirmed that insulin resistance initially emerges during mid-childhood, specifically within the first few years of puberty. The onset of insulin resistance was found to be more closely associated with childhood age than with puberty itself. However, over 60% of prepubertal insulin resistance variation remains unexplained [29]. Given the shifting demographics of childhood diabetes, it is controversial whether insulin resistance arises from the onset of puberty. Recently, a study conducted on healthy children aged 5–14 years revealed an escalation in insulin resistance from the age of 7 years onwards. During the initial three years of adolescence, the combined influence of obesity and elevated IGF-1 levels accounted for 34% of variations in insulin resistance among boys and 35% among girls, with IGF-1 exhibiting a more significant impact on insulin resistance in boys, despite of their relatively low IGF-1 levels [30].

Currently, it is widely acknowledged that leptin is involved in the regulation of pubertal development by exerting its effects on the hypothalamic–pituitary–gonadal (HPG) axis [31]. Upon reaching a specific threshold, adipose tissue-produced leptin binds to hypothalamic leptin receptors, thereby counteracting the inhibitory effect of neuropeptide Y (NPY) on hypothalamic gonadotropin-releasing hormone (GnRH) through suppression of NPY secretion. Consequently, this triggers the secretion and release of GnRH, promoting the synthesis and release of luteinizing hormone (LH) and follicle-stimulating hormone (FSH). When the body weight and fat stores increase with the growth and development of children, leptin produced by adipose tissue will reach a specific threshold and bind to leptin receptors in the hypothalamus, thereby counteracting the inhibitory effect of NPY on GnRH by inhibiting NPY secretion. Consequently, this triggers the secretion and release of GnRH, promoting the synthesis and release of LH and FSH, and the onset of puberty is initiated by an elevation in serum estrogen levels [32]. Furthermore, studies demonstrated that elevated levels of leptin in children with obesity may precipitate early onset of puberty [33]. During puberty, leptin suppresses insulin secretion from pancreatic *β* cells, but due to a transient decrease in insulin sensitivity and a compensatory increase in insulin secretion, insulin in turn promotes fat accumulation and leptin release, resulting in elevated levels of leptin in children [34]. Therefore, it is crucial to prevent the development of leptin resistance during puberty in children with obesity. Notably, dysregulation of leptin levels, whether excessive or deficient, can exert an impact on fertility. While gradual elevation of leptin levels in prepubertal males may facilitate testicular development, elevated serum leptin levels could also lead to lower sperm counts [34].

The timing of puberty onset is regulated by a combination of genetic, environmental, and social factors. Currently, it is widely acknowledged that the relationship between childhood obesity and puberty onset can be attributed to the regulation of the HPG axis through nutritional status and metabolic pathways [35, 36]. Most studies investigating the association between pediatric obesity and the timing of pubertal initiation have predominantly focused on females, revealing a positive correlation between obesity in girls and an earlier onset of puberty [37]. However, previous studies of obesity and pubertal onset in boys have produced inconsistent results, with some reporting an earlier onset of puberty while others suggesting a delayed onset among boys with obesity. During early puberty, a disparity in plasma leptin levels is observed between sexes, with females exhibiting significantly higher levels compared to males. This observation is consistent with the occurrence of early puberty in females and delayed sexual maturation in males [38, 39].

Obesity exerts not only a noticeable influence on the timing of pubertal onset in children but also significantly impacts the levels of pubertal hormones. During puberty, the HPG axis establishes a new equilibrium, wherein the maintenance of normal insulin sensitivity under physiological conditions is attributed to the presence of testosterone and estradiol. Conversely, elevated levels of estradiol or decreased levels of testosterone may contribute to the development of insulin resistance [40]. Insulin resistance triggers a compensatory elevation in circulating insulin levels, an augmentation in ovarian estrogen and adrenal androgen secretion, and a decrease in sex hormone-binding globulin concentration. Consequently, this leads to elevated steroid concentrations and bioavailability, thereby contributing to an earlier onset of puberty among children with obesity [34]. Compared to boys, girls may exhibit greater insulin resistance, which could be partially attributed to the effects of estrogen and progesterone. Previous studies have not established a significant association between elevated testosterone levels and insulin sensitivity in pubertal girls, nor have they implicated high estrogen levels in impaired insulin sensitivity among young women [23]. Recently, the elucidation of the involvement of both estrogen and progesterone in heightened insulin resistance in women has shed light on the intricate interplay between sex hormones and insulin [40].

## 4. Causes of Insulin Resistance in Individuals with Obesity

Currently, the mechanism underlying insulin resistance in obesity remains incompletely understood, and there is a lack of explicit literature identifying differences in the mechanisms of insulin resistance between adults and children. However, most individuals with insulin resistance are accompanied by obesity or overweight, a phenomenon that is gaining attention [41]. The dysfunction of adipose tissue and abnormal adipose metabolism are the primary etiological factors in obesity-related metabolic disorders. As the largest endocrine organ, the involvement of white adipose tissue in the development of T2DM and insulin resistance has been recognized. Moreover, the imbalance of redox reaction and the occurrence of oxidative stress (OS), mitochondrial dysfunction, inflammation, and genetic factors have been shown to be responsible for reduced insulin sensitivity.

### 4.1. Lipid Metabolism Disorders

As a consequence of obesity, the upregulation of hormone-sensitive lipase activity in adipose tissue resulted in an elevation of serum levels of FFA, diacylglycerol, and related metabolites. These metabolites were deposited into nonadipocytes, leading to a cascade of metabolic changes and abnormalities in cytokine expression and consequently inhibiting insulin signaling pathways and exacerbating insulin resistance [42].

#### 4.1.1. Free Fatty Acids

**Figure 1:**
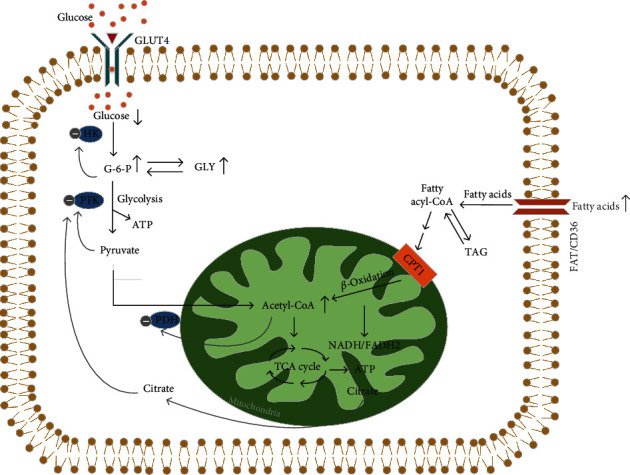
The competitive metabolism of glucose and fat: Upon glucose entry into the cell via glucose transporter 4 (GLUT4), two pathways are involved in the generation of G-6-P: glycogen (GLY) generation and oxidative energy production. Key enzymes such as PFK and pyruvate dehydrogenase (PDH) facilitate energy oxidation, but *β*-oxidation of FFA inhibits their activity, leading to decreased G-6-P oxidation and increased GLY synthesis as consequence. The accumulation of GLY inhibits the activity of hexose kinase (HK), which eventually leads to a reduced cellular glucose uptake.

FFA is a crucial energy source that is stored in adipose tissue as triglycerides. The potent inhibitory effect of insulin on lipolysis effectively suppresses the release of FFA from adipocytes. In children with obesity, adipocyte hypertrophy often leads to elevated serum FFA levels and accumulation of fatty acids in nonadipose tissues due to the weakening of insulin's antilipolytic effect [43]. Such ectopic fatty acids deposition can cause lipotoxicity and impair insulin sensitivity [44]. Considering the close relationship between fat and carbohydrate metabolism, alterations in plasma FFA levels and their metabolism are both a cause and consequence of insulin resistance and T2DM [45]. For instance, galectin-12, a protein preferentially expressed in adipocytes, has been found to be associated with a reduction in lipolysis, an increase in adiposity, and a deterioration of insulin resistance [46]. It was first proposed by Randle et al. that obesity-induced insulin resistance could be attributed to increased circulating FFA, competing with glucose for oxidative metabolism in insulin-sensitive cells, which is known as the glucose–fatty acid or Randle cycle, as illustrated in Figure 1. Fatty acids can upregulate the level of mitochondrial acetyl-CoA through *β*-oxidation in tissues including the liver, cardiac muscle, and skeletal muscle. The elevation of acetyl-CoA levels subsequently upregulates intracellular citric acid concentrations and inhibits phosphofructokinase (PFK), leading to an accumulation of glucose-6-phosphate (G-6-P) within cells, increased intracellular glucose levels, and decreased peripheral glucose uptake [47]. Upregulated acetyl-CoA also stimulates mitochondrial activity and activates OS-sensitive transcription factors such as nuclear factor-kappa (NF-*κ*B), a major mediator of inflammation. This leads to excessive production of reactive oxygen species (ROS). Recently, inhibition of NF-*κ*B activation by excess energy was found to improve mitochondrial dysfunction and insulin sensitivity in skeletal muscle, and FFA and its metabolites, such as acyl-CoAs or ceramides, can activate signaling protein kinases including protein kinase C (PKC), c-Jun N-terminal kinase (JNK), and I*κ*B protein inhibitor (IKKB) [48]. These kinases inhibit insulin signaling by promoting serine phosphorylation of insulin receptor substrate-1 (IRS-1) [49]. Furthermore, elevated levels of FFAs resulting from mitochondrial uncoupling and ROS production render children with obesity more susceptible to developing insulin resistance [49].

Several studies also demonstrated that ectopic deposition of diacylglycerol (DAG) contributes to the development of insulin resistance. By establishing a mouse model on a high fat ketogenic diet, Jornayvaz et al. discovered that this diet promoted energy expenditure and resulted in weight loss, however, it did not improve insulin resistance in the liver and was accompanied by a 350% increase in hepatic DAG levels [50]. Detection of childhood obesity through high insulin–glucose clamp testing found that elevated DAG levels can induce the translocation of PKC to the plasma membrane, thereby inhibiting insulin receptor tyrosine kinase (IRTK) activity through phosphorylation at Thr1160. Consequently, this leads to a decrease in insulin-stimulated phosphorylation of IRS-1, phosphoinositid 3-kinase (PI3K), and protein kinase B (PKB, also known as Akt), resulting in the blockage of insulin signaling [2, 51].

#### 4.1.2. Adipokines

In addition to energy storage, adipose tissue also serves as an active endocrine organ and plays a crucial role in cellular reactions and metabolic homeostasis. Adipokines, which are peptide hormones encoded by obesity genes and synthesized by adipose tissue, can modulate glucolipid metabolism, insulin sensitivity, and inflammatory response through autocrine, paracrine, or endocrine pathways [52]. Numerous adipokines have been identified nowadays, including acute-phase cytokines such as C-reactive protein (CRP), plasminogen activator inhibitor 1, haptoglobin, tumor necrosis factor-alpha (TNF-*α*), interleukin-6 (IL-6), IL-8, IL-10, transforming growth factor-beta (TGF-*β*), chemokines, CC-motif chemokine ligand 2 (CCL2), CCL5, and C-X-C motif ligand 8 (CXCL8). Among these, leptin, adiponectin, CRP, TNF-*α*, and resistin are closely associated with the occurrence and development of insulin resistance [53].

*(1) Leptin*. Leptin, a 16 kDa polypeptide encoded by the *leptin* (*LEP*) gene and produced by white adipose tissue, was initially discovered through positional cloning in 1994 by Friedman et al. [54]. The hormone leptin serves not only as a regulator of adiposity, but also functions as an anti-inflammatory cytokine closely linked to insulin resistance, playing a pivotal role in the regulation of weight loss and glucolipid metabolism [32]. Specifically targeting receptors in the hypothalamus, leptin is regarded as a key regulator of both satiety and circadian rhythms. Therefore, leptin-related signaling pathway may serve as a key mechanism linking sleep and obesity. It has been evidenced by a domestic population-based cohort study that six obesity-related genes, including *fat mass and obesity-associated protein* (*FTO*), *melanocortin-4 receptor* (*MC4R*), *mitogen-activated protein kinase 5* (*MAP2K5*), *glucosamine-6-phosphate deaminase 2* (*GNPDA2*), *proprotein convertase subtili*sin*/kexin type 1* (*PCSK1*), *and brain-derived neurotrophic factor* (*BDNF*), were strongly associated with sleep duration, increased leptin levels, and the risk of childhood obesity [55]. The regulation of leptin and insulin typically occurs bidirectionally. Insulin promotes the secretion of leptin, which in turn inhibits the insulin secretion through the negative feedback loop involving insulin, leptin, and NPY. Once this feedback is disrupted, the antagonistic effect of leptin on insulin production affects signal transduction pathways following insulin–receptor binding, resulting in the development of insulin resistance [56]. The mechanism by which leptin acts on insulin resistance involves the inhibition of gluconeogenesis through modulation of the autophosphorylation of IRTK, the tyrosine phosphorylation of PI3K, and the interaction between IRS-1 and growth factor receptor-binding protein 2 (GRB2), thereby impeding insulin signal transduction and attenuating its effects [1]. Furthermore, leptin can also promote lipolysis and increase serum FFA, thereby exacerbating insulin resistance [32].

The presence of hyperinsulinemia in individuals with obesity is typically accompanied by a significant elevation in serum leptin levels; however, impaired body responsiveness to leptin would lead to the inability of leptin to exert its normal physiological effects in peripheral tissues and the central nervous system, resulting in the development of leptin resistance. Besides insulin resistance, leptin resistance also contributes to the challenges faced by most obese in their weight loss efforts. Leptin resistance is typically associated with reduced expression of leptin receptors and inhibition of downstream signaling pathways, which are closely linked to the hypothalamic black cortex system, increased activity of matrix metallproteinase, ERS, and inflammatory response [57]. Earlier, hyperleptinemia was identified as a sufficient prerequisite for the induction of leptin resistance. It was hypothesized that in individuals with obesity, a decrease in serum leptin levels might restore hypothalamic leptin sensitivity and consequently lead to reduced weight gain [58]. Recent study conducted in a high-fat diet mouse model revealed that partial leptin deficiency prevented the upregulation of leptin levels, consequently leading to the protection of mice against diet-induced obesity and metabolic disorders [59]. The findings from these animal studies suggest that maintaining low leptin levels may be beneficial for management of obesity and diabetes, although its effectiveness needs to be clinically verified. So far, the underlying mechanisms of leptin resistance remain incompletely understood, with evidence suggesting that it is likely triggered by chronic inflammation-induced stress response and prolonged obesity-induced hyperleptinemia [60]. In summary, enhancing leptin sensitivity is deemed a crucial intervention for managing childhood obesity.

*(2) Adiponectin*. Like leptin, adiponectin is another adipose cytokine produced by adipocytes, skeletal muscle cells, cardio myocytes, and endothelial cells. In human circulation, adiponectin exists in a polymer form. Interestingly, ~90% of adiponectin exists in the form of high molecular weight (HMW) and low molecular weight (LMW) polymers, while only 10% exists as trimeric full-length adiponectin [61]. Investigation by Aso et al. has confirmed that HMW is a biologically active form of adiponectin, exhibiting various biological effects such as antidiabetes, antiatherosclerosis, and anti-inflammatory properties [62]. These effects are achieved through diverse mechanisms. For example, adiponectin binds to its specific receptor AdipoR2 in the liver, activating AMP-dependent protein kinase (AMPK) and increasing acetyl-CoA carboxylase phosphorylation. This promotes fatty acid consumption, inhibits liver gluconeogenesis, and reduces sugar output. In the liver and skeletal muscle, adiponectin enhances fatty acid oxidation via peroxidase proliferator-activated receptor alpha (PPAR-*α*), leading to decreased triglyceride levels and improved insulin sensitivity [61].

In contrast to leptin, serum adiponectin level decreases with weight gain, and there exists a negative correlation between leptin and adiponectin [63]. It has been shown by a clinical research that adiponectin level in children with obesity was lower than that in normal children, and was negatively correlated with triglyceride and HOMA-IR [64]. Hence, it could be speculated that insulin resistance in most children or adolescents with obesity may be related to decreased adiponectin level.

**Figure 2:**
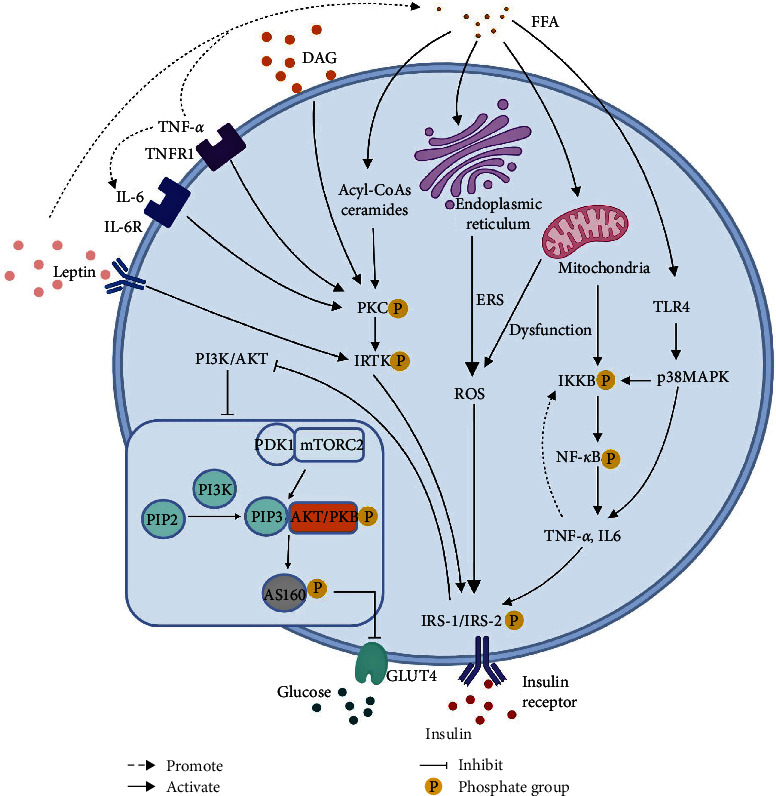
Mechanism of fat metabolite and adipocyte cytokines-induced insulin resistance in insulin targeting cells. It is shown that FFA, DAG, inflammatory factors (TNF-*α* and IL-6), and leptin promote IRS-1/IRS-2 phosphorylation by targeting IRS kinases such as p38 mitogen-activated protein kinase (p38MAPK), PKC, and ROS, thereby interfering PI3K-AKT pathway and inhibiting insulin signaling and consequent inhibition of cellular glucose uptake, in which mitochondrial dysfunction and endoplasmic reticulum stress (ERS) is involved.

*(3) Other Adipocyte Cytokines*. In children with obesity, FFA and adipokines (e.g., leptin) secreted by enlarged adipocytes promote the differentiation of monocytes to proinflammatory macrophages in adipose tissues, leading to the production of inflammatory cytokines including TNF-*α* and IL-6. With the combined effects of mitochondria, endoplasmic reticulum, and other cellular organelles, inflammatory cytokines contribute to the mechanism of insulin resistance primarily by interfering with the PI3K/AKT, and ROS pathways, as illustrated in Figure 2. As a signaling molecule, IKKB is also involved in TNF-*α* induced insulin resistance through two main pathways. Firstly, IKKB inhibits the insulin signaling pathway by phosphorylating serine residues of IRS-1 [65]. Secondly, it induces insulin resistance by activating NF-*κ*B and upregulating inflammatory factors such as TNF-*α* and IL-6 [66]. Molecular markers of inflammation, such as TNF-*α*, IL-6, and CRP, were significantly elevated in individuals with obesity and insulin resistance, and these markers could predict the development of T2DM [67]. Recent studies on T2DM and anti-inflammatory effects have shown the involvement of inflammatory reactions in the development of vascular lesions, especially large vessel lesions [68]. When vascular lesions occur, the levels of inflammatory markers such as CRP and IL-6 are elevated. As an important regulator of CRP, IL-6 induces insulin resistance and dyslipidemia, and is directly related to the consequences of vascular lesions [68]. Interestingly, besides immune cells such as monocytes, neutrophils, and natural killer (NK) cells, adipose tissue is also a significant source of endogenous TNF-*α* [53]. The level of plasma TNF-*α* has been shown to correlate with obesity severity and is linked to obesity-induced insulin resistance, and lipid-derived or myogenic TNF-*α* contributes to peripheral tissue insulin resistance induced by adiposity. Activated TNF-*α* can elevate plasma FFA levels by promoting lipolysis, inhibit the tyrosine kinase activity of insulin receptors in muscle tissue, and suppress IRS-1 phosphorylation and *GLUT4* gene expression, resulting in insulin resistance and also stimulate IL-6 production [69].

Resistin is well-known for its direct antagonistic effect on insulin. It has been discovered recently that resistin secreted by adipocytes can impede insulin-induced glucose uptake, in contrast, the rate of glucose uptake accelerated after neutralizing resistin [70]. Most studies investigating the relationship between resistin and insulin resistance propose that resistin is positively correlated with insulin resistance by inhibiting the IRS-1/PI3K/AKT pathway [49, 71]. In addition, resistin has been found to upregulate the expression of inflammatory factors such as TNF-*α*, IL-6, and interleukin-1beta (IL-1*β*) which can lead to abnormal insulin signal transduction by interfering NF-*κ*B inflammatory signaling pathway [52]. Research on the mechanisms of resistin and insulin resistance is ongoing. A recent study conducted by He et al. demonstrated that leucine-rich alpha-2-glycoprotein 1 (LRG1), an adipokine associated with obesity, inhibits hepatic insulin signaling by downregulating IRS-1 and IRS-2, thereby exacerbating high-fat diet-induced insulin resistance [72].

### 4.2. Chronic Inflammation Response

The chronic low-grade inflammatory state is also associated with insulin resistance [73]. The accumulation of macrophages in adipose tissue is considered to be a major source of local or systemic inflammatory mediators such as TNF-*α*, IL-6, and IL-1*β*. For one thing, these cytokines interfere insulin signal transduction and induce insulin resistance by activating suppressors of cytokine signaling (SOCS) in insulin target tissues and disrupting the insulin pathway at the level of insulin receptors or receptor substrates; for another, they interact with OS process to aggravate insulin resistance [74]. Moreover, inflammatory factors such as IL-1*β*, have been shown to exacerbate the development of T2DM by causing insufficient insulin secretion through inducing the deposition of islet amyloid and apoptosis in pancreatic *β* cells [75]. NK cells are considered to promote obesity-induced adipocyte stress by regulating macrophage recruitment and insulin resistance. In animal models, the absence of NK cells, natural cytotoxicity receptor 1 (NCR1), or interferon was found to impede macrophage recruitment in visceral adipose tissue, thereby enhancing insulin sensitivity [76]. Nevertheless, further studies are required to uncover more unknown pathways involved in this process.

As immune cells activated during chronic inflammation require increased glucose consumption for energy, insulin resistance may be induced by chronic inflammation, which raise blood glucose to meet the requirement [77]. Early study by Tobiume et al. demonstrated that apoptosis signal-regulating kinase1 (ASK1) could activate the JNK signaling pathway, which was responsible for autoimmune response or cellular stress leading to insulin resistance [78]. Later, it was reported that fatty acids could activate NF-*κ*B and p38MAPK signaling pathways through the upregulation of Toll-like receptor 4 (TLR4) expression in adipocytes and macrophages, thereby enhancing ERS, generating ROS and promoting secretion of proinflammatory cytokines [49]. NF-*κ*B is a major inflammatory regulator that controls transcription of numerous proinflammatory factors including TNF-*α*, IL-6, and CRP. Activation of NF-*κ*B inhibits the IRS-1/PI3K/AKT/GLUT4 pathway, impels oxidative energy supply, and aggravates insulin resistance. In turn, the aggravation of insulin resistance promotes an inflammatory response [79]. Chronic inflammation is not a direct and primary causative factor of insulin resistance, nor is it sufficient to disrupt systemic glucose metabolism. Nevertheless, it indirectly exacerbates insulin resistance and contributes to the progression of diabetes-related complications.

Prevalence of obesity is currently increasing across all ages, and the onset of secondary inflammation may occur during early stages of development [80]. The impact of obesity on metabolism and the immune system is lifelong, with varying cutoff points from prenatal stages to adolescence. Weight loss could normalize inflammatory markers in children with obesity, inspiring us to intervene in weight by altering inflammatory markers after the onset of inflammation [81]. In 2021, a meta-analysis comprising 27 randomized controlled trials demonstrated that participation in aerobic exercise among children and adolescents with overweight and obesity resulted in reduced BMI, fasting insulin levels, free fat mass, TNF-*α* and IL-6, and an improvement in physical fitness when compared to the control groups [82]. These findings suggested that aerobic exercise can be considered as a nonpharmacological intervention with potential benefits for reducing inflammation in individuals who are overweight or obese. In addition, a study conducted in China demonstrated that liraglutide and metformin treatment in obese individuals with primary T2DM inhibited the TLR4-NF-*κ*B signaling pathway and downregulated downstream molecules of the DAG/PKC signaling pathway, and controlling blood glucose levels or treating insulin resistance can effectively alleviate microinflammation [83]. In summary, the elucidation of the relationship between insulin resistance and inflammation provides information for treatment of insulin resistance and its related metabolic disorders such as T2DM and serves as a theoretical foundation for novel drug research and development.

### 4.3. Mitochondrial Dysfunction

Fat overload can cause oxidative damage of cellular proteins and nucleic acids, leading to mitochondrial dysfunction [84]. This dysfunction impairs the ability of mitochondria to integrate metabolic signals triggered by glucose, ultimately reducing reactive insulin secretion of islet *β* cells in response to glucose stimulation [85]. Mitochondrial dysfunction might be a consequence of proinflammatory signaling and OS, both of which are hallmarks of metabolically complex obesity. Once mitochondrial dysfunction occurs, it would exacerbate OS, insulin resistance, and inflammation by promoting the production of ROS. Moreover, impaired mitochondrial fatty acid oxidation may worsen lipotoxicity and fatty acid accumulation, inhibiting the PI3K/AKT signaling pathway and leading to insulin resistance [86]. In consideration that OS is a predisposition factor for insulin resistance, rectifying the imbalance between ROS and antioxidants may ameliorate insulin resistance [87]. JNK, p38MAPK, and IKKB are the primary kinases activated by OS that result in serine phosphorylation of IRS-1 and IRS-2. This, in turn, impedes insulin signaling by inhibiting tyrosine phosphorylation of IRS. Increased mitochondrial ROS and downregulated mitochondrial antioxidant enzymes were found in obese patients with T2DM compared to normal individuals. Yimei et al. reported that mitochondrial impairment could be restored by daily exercise or treatment with the insulin sensitizer rosiglitazone [88]. Since rosiglitazone is not currently approved by the Food and Drug Administration (FDA) for the treatment of T2DM in children and adolescents, increasing physical activity remains a viable intervention option.

### 4.4. Endoplasmic Reticulum Stress

The accumulation of misfolded or unfolded proteins within the endoplasmic reticulum can trigger ERS. To cope with and regulate ERS, as well as maintain endoplasmic reticulum homeostasis, cells have evolved an evolutionarily conserved mechanism known as the unfolded protein response (UPR) [89]. Once the stress becomes chronic and surpasses the adaptive capacity of UPR, it triggers either apoptosis or ERS-mediated cell death. Meanwhile OS is exacerbated, which in turn leads to further impairment of endoplasmic reticulum function. These processes have been implicated in a variety of serious diseases including neurological disorders, cardiovascular disease, diabetes, and ischemia reperfusion injury [90]. To date, the mechanism underlying obesity-induced ERS has not been completely identified. The phosphorylation and activation of specific ERS markers within adipose tissue in obese mice indicated that this location serves as a primary site for obesity-induced ERS [91]. Moreover, recent findings have suggested that calreticulin and protein disulfide isomerase family A member 3 (PDIA3), two markers for ERS, were associated with insulin resistance induced by childhood obesity. Therefore, ERS has been considered as a hub linking obesity, insulin resistance, and T2DM [92].

The onset of ERS triggers the phosphorylation of inositol-requiring enzyme-1*α* (IRE1*α*). Through the recruitment of ASK1 and tumor necrosis factor receptor-associated factor-2 (TRAF-2), these three proteins form a complex that jointly activates cJUN-N-terminal kinase and serine kinase, leading to the phosphorylation of serine residues on IRS-1. Thereby it could inhibit the PI3K-mediated insulin signaling pathway and reduce insulin sensitivity in target tissues [93]. Besides, the over activation of JNK during sustained ERS promotes serine phosphorylation and inhibits tyrosine phosphorylation of IRS-1, which consequently causes inhibition of insulin receptor signaling and insulin resistance [91, 93]. Currently, it has been observed that ERS-induced insulin resistance may not depend on the JNK pathway, but on the downregulation of insulin receptor expression via autophagy pathway [94, 95]. Moreover, inhibitor factor-kB (IkB), mammalian rapamycin target protein, and PKC zeta were also involved in serine phosphorylation of IRS-1 protein [96].

### 4.5. Serum Uric Acid

The incidence of hyperuricemia among children is increasing annually due to changes in lifestyle and diet, resulting in elevated serum uric acid (SUA) levels. The early onset of hyperuricemia poses a more significant threat to the health of children and adolescents, and besides kidney disease, it increases the risk of developing hypertension and hyperlipidemia in adulthood [97]. By collecting and analyzing data of SUA, BMI, abdominal circumference, and HOMA-IR, Fu et al. discovered a significant association between elevated SUA in children with obesity and the degree of insulin resistance [98]. The presence of increased visceral fat content leads to abnormal purine metabolism and elevated levels of SUA, thereby promoting the deposition of FFA within the body and inducing insulin resistance [98]. Additionally, high SUA levels could also inhibit glycolysis by inactivating glyceraldehyde-3-phosphate dehydrogenase, resulting in elevated blood glucose levels and prone to insulin resistance [99]. In experimental mice, an elevated level of SUA was found to inhibit the IRS1-PI3K-AKT signaling pathway and trigger insulin resistance, suggesting the involvement of ROS in SUA-induced insulin resistance [100]. The primary mechanism involves the elevation of SUA concentration, which causes the deposition of uric acid crystals within pancreatic islets, leading to pancreatic *β* cells damage and subsequent insulin resistance [100]. Hyperuricemia may also inhibit insulin signal transduction by stimulating leukocytes to release inflammatory factors, yet its specific signaling pathway needs further investigation.

Hyperuricemia may serve as an independent predictor of insulin resistance, and insulin resistance in turn promotes the development of hyperuricemia [101]. Therefore, in children and adolescents, weight management and reduction of consumption of high-purine and high-fructose diets while ensuring normal growth and development, attention to joint and renal pathology, and timely adjustment of therapeutic strategies can help to prevent or minimize the occurrence of hyperuricemia [102].

### 4.6. Genetic Factors

#### 4.6.1. Obesity-Related Genes

The involvement of genetics in the pathogenesis of childhood obesity has been well-documented. Single gene mutations, genetic polymorphisms, and epigenetic inheritance interact with environmental factors to contribute to the development of childhood obesity. In 2007, Frayling et al. discovered that the rs9939609 variant of the *FTO* gene exhibited a strong association with obesity in both adults and children, thereby initiating an up-surge of genome-wide association studies (GWAS) of obesity [103]. To date, over 300 genes have been identified as closely related to obesity, such as *leptin receptor* (*LEPR*) gene, *pro-opiomelanocortin* (*POMC*) gene, *LEP* gene, *FTO* gene, *neuropeptide Y* (*NPY*) gene, and *melano-cortin-4-receptor* (*MC4R*) gene, etc. These human obesity-related genes are predominantly located on the chromosomes 2, 7, 16, 17, and 18 and play a significant role in regulating energy intake and consumption within the body [104]. Numerous studies have demonstrated that the minor T allele (TaqIA1) is associated with a reduced density of dopamine D2 receptors (DRD2) within the brain's dopamine pathway, and individuals carrying *TaqIA1* variant with impaired DRD2 function exhibit significant weight gain following episodes of overeating [105, 106]. TaqIA1 is also found to be associated with the *FTO* gene, which regulates the dopamine pathway implicated in obesity and insulin resistance.[107] Moreover, the activation of *MC4R* has been demonstrated to induce a decrease in food intake and an increase in energy consumption [108] and the *MC4R* rs17782313 variant has been identified as a predisposing factor for obesity in offspring, and associated with insulin resistance, dyslipidemia, and an increased risk of T2DM as well [109]. Additionally, the *MC4R* rs17773430 variant was found to be positively associated with an increased percentage of body fat mass [110]. Currently, there is a paucity of GWAS conducted in pediatric populations. In 2019, a large trans-ancestral meta-analysis of GWAS identified 19 single nucleotide polymorphism (SNP) loci associated with childhood obesity (*methyltransferase-like 15* (*METTL15*) rs10835310, *SEC16 homolog B* (*SEC16B*) rs539515, *BDNF* rs17309874, *troponin I-interacting kinase* (*TNNI3K*) rs10493544, *adenylate cyclase 3* (*ADCY3*) rs4077678, etc.) [111]. A study conducted on Mexican children revealed a significant correlation between 11 SNP loci (*SEC16B* rs543874, *olfactomedin 4* (*OLFM4*) rs12429545, *FTO* rs9939609, *MC4R* rs6567160, *GNPDA2* rs13130484, etc.) and the BMI of children. Additionally, 7 SNP loci (*SEC16B* rs543874, *OLFM4* rs12429545, *FTO* rs9939609, *MC4R* rs6567160, *GNPDA23* rs1330484, etc.) were found to be associated with pediatric obesity [112]. A Spanish cohort study demonstrated that C-C-C haplotype at three SNP loci (rs17782313, rs17773430, and rs34114122) within the *MC4R* gene significantly augmented early metabolic risk factors in children with obesity [113]. The findings from studies conducted in different countries regarding the correlation between SNPs in obesity-related genes and BMI as well as the risk of obesity in children exhibit inconsistencies, implying the influence of regional and racial disparities in genetic variations associated with obesity. Moreover, these disparities may also impact the development of obesity through interactions with other genetic or environmental factors.

#### 4.6.2. Epigenetic Modifications

DNA methylation, a vital component of epigenetic modifications, represents one potential mechanism that underlies obesity or T2DM [114]. A study conducted by Wahl et al. demonstrated a significant association between DNA methylation and obesity, highlighting its potential as a predictive tool for future development of T2DM [115]. The risk of obesity in offspring may be increased by environmental factors such as parental prepregnancy BMI, maternal gestational BMI, and intrauterine nutritional status through DNA methylation early in life [116, 117]. The high methylation of the *insulin-like growth factor 2* (*IFG2*) *and H19* genes (*IGF2/H19*), *serotonin transporter gene* (*SLC6A4*), *retinoid X receptor alpha* (*RXRalpha*), *endothelial nitric oxide synthase* (*eNOS*), and *trophoblast Cell Surface Antigen 2* gene (*TACSTD2*) at birth have been linked to subsequent obesity development, suggesting that locus-specific DNA methylation is established early in life but exerts its effects later on. These identified loci are potential for early prediction of obesity risk [118, 119, 120, 121]. Among them, *IGF2/H19* is involved in the pathogenesis of obesity-related diseases such as diabetes mellitus, and evidence suggested that parental obesity impacts the DNA methylation of *IGF2* in offspring [122].

The comparison of genome-wide DNA methylation profiles between children with and without obesity has revealed a significant number of differentially methylated loci. However, only a limited subset of these loci has shown consistent associations with obesity development after rigorous multiple corrections or validation in different populations, for instance, *lymphocyte antigen 86* (*LY86*) methylation and *single-minded homolog 1* (*SIM1*) methylation [123]. The *LY86* gene encodes the lymphocyte surface antigen, and subsequent validation in diverse populations has revealed an association between *LY86* methylation and the occurrence of obesity as well as insulin resistance, suggesting its potential involvement in the development of obesity-related metabolic disorders [124]. The study conducted by Bonnefond et al. demonstrated a strong correlation between loss-of-function *SIM1* mutations, severe obesity, and the presence of Prader–Willi-like syndrome (PWL) features in a cohort of 1,193 children [125]. Recently, a significant association has been discovered for the first time between increased methylation level at the Chr6: 100903612 locus in *SIM1* and children's BMI [126]. Although there are preliminary data available that reveal genome-wide obesity-related DNA methylation patterns in children, further studies are necessary to determine the levels of DNA methylation at specific loci of obesity-related genes.

Epigenetic modifications also contribute to the pathogenesis of T2DM by regulating pancreatic development and *β* cells function, and the molecular mechanisms involve alterations in DNA methylation, histone methylation and acetylation, and noncoding RNA (ncRNA) [114]. The development of diabetes can be influenced by environmental factors through the modulation of DNA methylation, which promotes the initiation of inflammatory responses and interferes with insulin secretion, and these effects may even be inherited by offspring. Chen et al. observed that a hyperglycemic environment leads to a reduction in the expression level of maternal effector tet methyl cytosine dioxygenase 3 (TET3) in female mouse oocytes, resulting in impaired DNA demethylation during early postfertilization embryonic development and causing abnormal hypermethylation of gene promoters involved in the insulin secretion pathway. The persistence of this aberrant hypermethylated state into adulthood can result in insufficient insulin secretion by offspring mice due to the inhibition of gene expression, thereby contributing to the intergenerational inheritance of susceptibility to diabetes mellitus [127]. Internationally, GWAS have successfully identified over 100 susceptibility genes for T2DM, such as *adenylyl cyclase 5* (*ADCY5*), *hematopoietically expressed homeobox* (*Hhex*), *Potassium voltage-gated channel subfamily Q member 1* (*KCNQ1*), and *cyclin dependent kinase inhibitor 2A* (*CDKN2A*). However, the individual SNPs associated with these genes only exhibit modest effects and do not serve as robust genetic predictors [128]. Therefore, the systematic development of genetic risk assessment models can provide novel insights for early identification of high-risk populations and timely intervention in disease. The research findings have demonstrated that histone modifications play a crucial role in the regulation of gene expression in pancreatic *β* cells through the recruitment of protein complexes, thereby exerting influence on their differentiation, proliferation, and apoptosis [129]. A series of vitro experiments have demonstrated that histone demethylation and histone deacetylation can contribute to the development of T2DM by inhibiting the development of pancreatic *β* cells, suppressing the expression of genes associated with the insulin secretion pathway, and inducing inflammatory responses [130, 131].

ncRNAs also play a crucial role involved in the regulation of insulin sensitivity. Among them, microRNAs (miRNAs), which are small ncRNAs, have been shown to modulate the expression of key proteins in the insulin signaling pathway, thereby affecting insulin sensitivity. This opens up promising avenues for exploring therapeutic strategies of T2DM, such as the miR-183 family, the miR-29 family, miR-27a, and miR-143 [132, 133, 134, 135]. Currently, circulating miRNAs are widely acknowledged as reliable predictive markers of insulin resistance in patients with obesity and hold significant potential for serving as predictors for the development of various obesity-related complications, including T2DM and cardiovascular disease [136, 137]. Using quantitative PCR (qPCR), Can et al. conducted a study to quantify circulating miRNAs in lean children and those with obesity, revealing that miR-335, miR-143, and miR-758 exhibited lower expression levels while miR-27, miR-378, and miR-370 showed higher expression levels in the group with obesity compared to the lean group [138]. Santos et al. categorized the subjects into a normal weight group based on BMIz and an overweight or obese groups for the study. They demonstrated that only miR-18a-5p was upregulated in children belonging to the overweight or obese groups, while miR-146-5p, miR-423-3p and miR-152-3p were found to be associated with insulin resistance irrespective of their insulin sensitivity [137]. Additionally, it has been demonstrated a significant association between altered levels of circulating miR-486-5 p, miR-146b-5p, and miR-15b-5p and both BMI and abdominal fat mass in children aged 4–6 years with obesity [139]. Notably, the study of circulating miRNAs in obesity is still in its early stages, and there is some variability in the findings of studies on circulating miRNAs in lean and obese children. This variability may be attributed to differences in methodologies, platforms, and products utilized by various vendors. With the advancement of research, it is anticipated that future investigations will successfully elucidate the underlying mechanisms of epigenetic involvement in diseases such as obesity and T2DM, and thus reverse or decelerate disease progression.

### 4.7. Others

Children with obesity often experience a decline in vitamin D levels and increasing the intake of vitamin D has been found to be effective in reducing the incidence of T2DM [140, 141]. The deficiency of Vitamin D exacerbates insulin resistance through direct inhibition of insulin synthesis and secretion, and also increasing the risk of insulin resistance by affecting fat metabolism, inflammatory response, OS, and mitochondrial function, which ultimately contribute to the development of childhood obesity. [142] Thus, Vitamin D supplementation may promote insulin secretion by inhibiting systemic inflammatory response, reduce insulin resistance, and beneficial to blood glucose control. High-fat diets and high-carbohydrate diets are strongly associated with the development of obesity. Significantly, high-fat diet-induced metabolic disorders are directly correlated with the development of insulin resistance, regardless of the children's obesity status [143]. Additionally, intestinal flora imbalance plays an important role in lipid metabolism disorders induced by high-fat diets. Wang et al. observed that a disturbance of intestinal flora in mouse models fed with a high-fat diet resulted in a reduction of short chain fatty acids (SCFAs), and activation of the intestinal flora-butyrate-insulin resistance pathway [144]. Moreover, hypoxia-inducible factor-1*α*, ethnicity, and gender are additional factors that may impact insulin sensitivity in children with obesity, particularly those with a family history of T2DM. The incidence and prevalence of T2DM in children and adolescents has significantly increased over the past decade, with intricate influences from psychosocial and cultural environments, as well as the aforementioned mechanisms. All these pose challenges to maintaining healthy lifestyles and self-management behaviors [145].

## 5. Chronic Complications of Childhood Obesity

The progressive nature of childhood obesity confers a 50%–75% likelihood of persisting into adulthood, thereby exerting detrimental effects on psychology and intelligence. Chronic complications associated with obesity mainly encompass T2DM, MS, nonalcoholic fatty liver disease (NAFLD), hypertension, cardiovascular disease, and obstructive sleep apnea. These comorbidities significantly augment the risk of treatment failure for obesity and premature mortality in adulthood [1].

T2DM in children is characterized by hyperglycemia, presenting with symptoms of thirst, polyuria, and weight loss, and the onset of diabetic ketoacidosis (DKA) accounts for ~6% of individuals aged 10–19 years with T2DM [12]. Similarly, insulin resistance is one of the pathogenic mechanisms of MS, and clinically manifested as hypertension and abnormal glucose/fat metabolism. A cross-sectional study has revealed that children and adolescents suffering from obesity are often associated with MS, and a higher risk of cardiovascular disease in adulthood [146]. Furthermore, a multicenter cross-sectional study revealed that children and adolescents with a BMI ranging from the 25th to 84th percentile exhibited an elevated risk of hypertension with their increased BMI [147]. The current prevalence of NAFLD in American children makes it the most common cause of chronic liver disease, leading to hepatic and extrahepatic conditions such as fatty liver, cirrhosis, and liver failure that can persist from childhood into adulthood [148]. As a reversible disease, early diagnosis and prompt treatment of NAFLD can lead to rapid resolution of clinical symptoms. Of notice, polycystic ovary syndrome (PCOS) is frequently observed in adolescents with insulin resistance; this may be attributed to the excessive production of ovarian androgens induced by hyperinsulinemia [149]. In addition, central obesity exerts a more pronounced influence on blood lipid levels in children compared to general obesity, and specific adipose tissue depots (visceral and subcutaneous) may increase cardiac risks in children and adolescents. Statistically, up to one-third of children and adolescents with obesity are suffering from obstructive sleep apnea, 6 times more often than the lean ones, and hypercapnia resulting from obstructive sleep apnea has been shown to impair hypothalamic gonadotropin-releasing hormone function and delay puberty [150]. Presently, the prevention and control of childhood obesity are urgent, especially in middle-income countries. It is imperative to implement targeted policies for efficient allocation of medical resources and reduction of premature mortality resulting from chronic complications associated with obesity [151].

## 6. Treatment of Insulin Resistance in Pediatric Obesity

### 6.1. Lifestyle Modification

Diet and physical exercise are the primary interventions for pediatric obesity, with dietary intervention emphasizing healthy dietary patterns that prioritize high quality foods while reducing calorie dense options, particularly sugar-containing beverages. The Portuguese government has posited that regular consumption of sugary drinks in children leads to weight gain, and thus introduced a sugary drink tax. It has been estimated that the impact of this policy on population diet surpasses the combined effects of all educational and self-regulatory mechanisms [152]. Moreover, the calorie intake should be based on maintaining a standard body weight, correcting metabolic disorders, and alleviating the burden on pancreatic islets *β* cells [153]. A recent umbrella review has demonstrated that a high consumption of Mediterranean and Dietary Approaches to Stop Hypertension (DASH) diets can significantly improve insulin resistance and reduce the risk for T2DM, particularly in individuals with a high susceptibility [154]. Dietary patterns also play a role in improving childhood obesity and insulin resistance. An experiment in mice has shown that excessive salt consumption could activate the aldose reductase-fructokinase pathway in the liver and hypothalamus, leading to endogenous fructose production, leptin resistance, and hyperactive appetite, and ultimately resulting in insulin resistance, obesity, and hepatic steatosis [155]. Therefore, restricting high-salt diets is beneficial to improve insulin resistance and imperative in children with obesity. Additionally, time-restricted feeding is a precise and feasible nutritional interference to ameliorate metabolic health, such as hepatic steatosis. Isocaloric intermittent fasting (IF) has been reported to improve both glucose tolerance and postprandial insulin levels as well as increase cretin expression in leptin-deficient *ob/ob* mice, suggesting a potential strategy for weight management [156].

Physical exercise is another key factor in the management of obesity with T2DM among children and adolescents. Adipose tissue hypoxia resulting from obesity can lead to metabolic disorders, and exercise can effectively alleviate this condition by improving oxygenation in adipose tissue, enhancing insulin sensitivity, and promoting metabolism of sugar and lipids. During exercise, skeletal muscle not only utilizes lipids as an energy source but also consumes glucose, which accelerates the uptake and utilization of FFA [157]. In addition, exercise training has been shown to ameliorate insulin resistance and augment glucose utilization and myogenic glycogen storage through upregulation of *GLUT4* expression [158]. Additionally, physical exercise can suppress chronic inflammation in adipose tissue by increasing blood flow and ameliorating the hypoxic microenvironment, which also contribute to enhancing insulin sensitivity [159]. A meta-analysis has demonstrated that maintaining a healthy lifestyle at the age of 4 can significantly reduce the risk of developing obesity at the age of 7, and the combination of diet intervention and physical exercise can improve HDL cholesterol, fasting insulin, and fasting blood glucose levels [160]. Therefore, children with T2DM are encouraged to engage in moderate to vigorous physical exercise for at least 60 min every day and reduce sedentary behavior.

### 6.2. Pharmacotherapy

#### 6.2.1. Weight-Loss Drugs

Drugs and bariatric surgery have become alternatives for the treatment of childhood obesity, as not all cases of overweight or obesity in children and adolescents can be effectively managed through diet and lifestyle interventions. However, limited drugs have been evidenced to be effective and authorized by the FDA. Currently, the only FDA-approved drugs for adolescents are liraglutide, orlistat, and phentermine/topiramate. These drugs have been shown to effectively improve glycemic control in T2DM patients and attenuate the progression towards T2DM in high-risk populations [12].

Liraglutide has recently been recommended for use in adolescents aged 12–17 years as an adjunctive therapy to lifestyle modifications. Glucagon-like peptide-1 (GLP-1) analog, a hormone involved in the regulation of appetite and food intake, binds to GLP-1 receptors on pancreatic *β* cells. This binding stimulates insulin secretion, enhances insulin sensitivity, reduces blood glucose levels, and suppresses hunger and energy intake [161]. Orlistat was FDA-approved in 2003 for the management of obesity in adolescents aged 12–16 years. Functioning as a lipase inhibitor, it effectively reduces fat absorption by up to 30% within the gastrointestinal tract [162, 163]. In terms of glucose metabolism, orlistat has been demonstrated to increase fasting plasma insulin levels and HOMA-IR in patients with obesity combined with T2DM and PCOS, both in short-term treatment and slightly prolonged courses. The metabolic improvements observed in obese or overweight patients treated with orlistat are mainly attributed to its inhibition of intestinal lipid absorption [164]. Notably, administration of orlistat capsules may lead to acute hepatic and renal injury as well as gastrointestinal adverse reactions, which imposes certain limitations on its application in adolescents [165].

Recently, the FDA has approved phentermine/topiramate extended-release capsules as an adjunct to a low-calorie diet and increased physical activity for chronic weight management in pediatric patients [166]. Phentermine may reduce appetite by modulating catecholamine release in the hypothalamus, while topiramate may enhance satiety and suppress appetite. However, the safety and efficacy of phentermine/topiramate in combination with other weight loss products (including herbal preparations, over-the-counter medications, and prescription drugs) have not been established, and its impact on major cardiovascular adverse events in pediatric patients has not been studied. The most common adverse reactions observed in children are neurological symptoms, such as depression and dizziness, followed by joint pain and fever [167].

Semaglutide, a novel long-acting glucagon-like peptide-1 receptor agonist (GLP-1RA), has been proposed by the FDA for the treatment of obesity in adults. By binding to the GLP-1 receptor, semaglutide promotes insulin synthesis and secretion, and plays an essential role in the central nervous system [168]. A study assessing the impact of semaglutide on weight loss in adolescents aged 12–17 years with obesity demonstrated that, when combined with diet and lifestyle management, treatment for 68 weeks resulted in an average decrease of BMI by 16.1% among individuals receiving semaglutide, while the placebo group experienced an average increase in BMI by 0.6%. However, the incidence of serious adverse events was slightly higher in the semaglutide group compared to the placebo group (11% vs. 9%). The semaglutide group exhibited a higher incidence of gastrointestinal discomfort, with nausea, vomiting, and diarrhea being the most common adverse reactions observed. Furthermore, preclinical studies conducted on rodents have indicated a potential association between semaglutide administration and an increased risk of thyroid tumors [169]. Therefore, individuals with a medical history of medullary thyroid carcinoma are contraindicated from semaglutide administration.

With the endeavors of researchers, an increasing number of drugs have emerged recently. Carmo-Silva and Cavadas [170] have discovered that octreotide, which is commonly used in the treatment of hypothalamic obesity, exerts an inhibitory effect on insulin and promotes stabilization of body weight and BMI [170]. Additionally, the administration of leptin has been found to significantly reduce fat mass in individuals suffering from leptin deficiency [32, 170]. Since adolescents with obesity are still in the stage of growth and development, compared to weight loss drugs used in adults, drugs for adolescents with obesity needs to be administrated more cautiously. Unfortunately, there is currently a lack of medication references for children under 12 years old. With the development of pharmacotherapy studies, it is expected that safer and more effective drugs will become available for children with obesity in the future.

#### 6.2.2. Noninsulin Drugs

Pharmacological options for treating T2DM in adolescents are currently limited to insulin, metformin, and GLP-1RA. Metformin is recommended as a first line therapy when initial insulin treatment is not required. The Type 2 Diabetes in Adolescents and Youth (TODAY) consortia study revealed that metformin alone was able to achieve more permanent blood glucose control in approximately half of the subjects. Meanwhile, in the investigation of lifestyle and metformin treatment in young individuals with T2DM, the combined therapy does not confer a more pronounced advantage in blood glucose control compared to metformin monotherapy [171]. Metformin alone is unlikely to pose a risk of hypoglycemia and the incidence of lactic acidosis is extremely low. There are some gastrointestinal adverse effects of metformin, such as nausea, transient abdominal pain, and diarrhea, and prolonged use of metformin is associated with vitamin B12 deficiency, therefore, regular monitoring of vitamin B12 levels should be considered [12].

For individuals who have utilized insulin, the 2022 clinical practice recommendations of the American Diabetes Association (ADA) advocate combining GLP-1RA treatment to attain superior and enduring therapeutic outcomes. Liraglutide is the second noninsulin medication approved for treating pediatric T2DM, following metformin's approval in 2000. A randomized clinical trial demonstrated that the subcutaneous administration of liraglutide in combination with metformin (with or without basal insulin) safely and effectively reduced Hemoglobin A1c (HbA1c) levels in patients aged 10–17 years with T2DM, albeit at an increased incidence of gastrointestinal adverse reactions [172]. Although liraglutide and exenatide have been recommended by the FDA for the treatment of T2DM in children over 10 years old, it is noteworthy that the concomitant use of liraglutide with insulin or other insulin secretagogues (e.g., sulfonylureas) may increase the risk of hypoglycemia, particularly in children [173]. In general, the use of FDA-unapproved drugs for adolescents with T2DM outside the scope of research trials is not recommended.

#### 6.2.3. Insulin

Basal and prandial insulin have been shown to be effective in the treatment of childhood obesity with T2DM [98]. For children with blood glucose levels ≥13.9 mmol/L, HbA1c levels ≥8.5%, and presenting typical diabetic symptoms (polyuria, polydipsia, and weight loss) but without DKA, combination therapy of basal insulin and metformin was preferred, and the dosage should be adjusted based on patient's condition [174]. The administration of basal insulin not only reduces the incidence of hypoglycemia and improves treatment adherence in children, but also mitigates postprandial and nocturnal hyperglycemia. On the one hand, insulin provides more rapid metabolic control compared to metformin; on the other hand, it preserves or even reverses *β*-cell damage. For children experiencing DKA, prompt administration of subcutaneous or intravenous insulin is imperative to rapidly correct hyperglycemia and metabolic disorders [175]. The findings of a study examining the utilization of insulin pumps in pediatric and adolescent populations indicated that the omission of merely two prandial insulin injections per week could result in a 0.5% increase in HbA1c levels. Currently, hypoglycemia remains the primary adverse effect of insulin, and the potential for weight gain should also be considered. Since T2DM is a progressive disease, many patients eventually need to be treated with insulin [176]. In adolescence, there is an anticipated increase in insulin requirements and adjustments to basal insulin strategies are also needed. In general, pediatricians can collaborate closely with knowledgeable endocrinologists, monitor pediatric glucose trends vigilantly, and adjust insulin dosages accordingly, all of which are essential for achieving optimal glycemic control.

#### 6.2.4. Traditional Chinese Medicines (TCM)

TCM has been used for treatment of obesity for a long time. TCM herbs and herbal formulas contain active molecules have been demonstrated to reduce body weight by downregulating leptin levels and improving insulin resistance [175]. It has been shown recently that celastrol could ameliorate insulin resistance and improve insulin sensitivity, mitigate renal and hepatic injury, and promote weight loss in diabetic mice [177]. Although previous studies have demonstrated the potential of celastrol in preventing and treating diabetes, the specific molecular mechanisms underlying its hypoglycemic effects remain unclear [178]. A recent study has indicated that the Erchen decoction and its modified formula could regulate glucose and lipid metabolism by modulating signal pathways related to lipid metabolism [179]. Interestingly, prescriptions containing lotus leaf, astragalus, and fructus crataegi, which intended to promote blood circulation, exhibit significant regulatory effects on serum leptin levels [180].

The plasticity of adipocytes has been found to be regulated by activating brown adipose tissue (BAT) and triggering browning of white adipose tissue (WAT). Currently, several promising browning agents derived from plant extracts or herbs that activate BAT and induce WAT browning, such as resveratrol, berberine, and curcumin, have demonstrated great potential in laboratory studies. For instance, resveratrol in polygonum cuspidatum can activate the silent information regulator sirtuin 1 (SIRT1) signaling pathway, leading to an increase in lipolysis rate, mitochondrial thermogenesis, and uncoupling protein 1 (UCP1) protein expression [181]. Moreover, tea saponin and ginsenoside were found to decrease body weight and suppress appetite. They also increase hypothalamic phosphorylated signal transducer expression and activator of transcription 3 (STAT3) and pro-opiomelanocortin in mice fed with a high-fat diet, and simultaneously enhancing insulin resistance sensitivity [182]. Due to the intricate and perplexing nature of the specific components and mechanism of action of TCM, further research is necessary to fully comprehend their overall role in metabolism and other physiological processes. According to the ADA 2022 guidelines, there is insufficient high-quality evidence to support the effectiveness of dietary supplements in managing childhood obesity or promoting weight loss, including TCM and herbal remedies [12]. However, these recent findings do provide new strategies for the management of T2DM and childhood obesity.

#### 6.2.5. Probiotics

Numerous previous studies have established a strong correlation between alterations in the gastrointestinal microbiota and the prevalence of obesity. The gut microbiota of children with obesity demonstrates a reduction in diversity and an increase in the abundance of oxygen-tolerant bacteria [183]. Probiotics exert their influence on body weight reduction by promoting the growth of specific dominant flora in the intestinal tract, regulating adipocyte metabolism directly, improving the low inflammatory state of the body, and regulating immunity. Probiotics are broadly defined as live nonpathogenic microorganisms, utilized to improve the microbial balance in the host, while in a narrow sense they specifically refer to food-grade strains such as *Bifidobacterium*, *Lactobacillus*, *Lactobacillus rhamnosus*, *Saccharomyces cerevisiae*, and *Lactobacillus casei* [184, 185].

*Bifidobacterium* establish early colonization in the human intestine and are integral members of the natural intestinal microbiota, contributing to beneficial effects by preventing infection from other bacteria (e.g., *Escherichia coli*), promoting carbohydrate and dietary fiber digestion, reducing lipopolysaccharide levels within the intestine, and fortifying intestinal barrier function [186]. The majority of studies so far have demonstrated a negative correlation between *Bifidobacterium* and T2DM, while several investigations have suggested that the involvement of *Lactobacillus* in the regulating host glucose metabolism may be synergistically related to *Bifidobacterium*. Therefore, *Mycobacterium spp*. and *Bifidobacterium* are considered beneficial genera for adjuvant treatment of T2DM [187, 188, 189]. *Lactobacillus* also play a beneficial role in regulating body fat percentage and maintaining glucose homeostasis. Certain strains of *Lactobacillus* and *Akkermansia muciniphila* demonstrate potent inhibition of *α*-glucosidase activity, effectively preventing the breakdown of complex carbohydrates and controlling postprandial hyperglycemia [190]. Other probiotics, such as *Bifidobacterium*, *L. acidophilus*, *Lactococcus lactis*, and *L. casei* exhibit hypocholesterolemic effects. In addition, Rastelli et al. revealed that probiotic fermentation products could promote WAT in obese mice by upregulating the expression of thermogenesis-related genes and proteins and reduce lipid ectopic accumulation in liver tissue [191].

As mentioned above, probiotic supplementation has been shown to effectively improve the balance of gastrointestinal microecology and holds clinical implications for the treatment of obesity and related metabolic diseases. However, most studies supporting the use of probiotics for obesity management are based on laboratory research, further investigation is required to identify specific probiotic species, modes of administration, and their intervention mechanisms in childhood obesity.

### 6.3. Metabolic and Bariatric Surgery (MBS)

Over the past decade, there has been a growing trend in performing MBS on adolescents with obesity. The Pediatric Weight Loss Study Group and the Teen-Longitudinal Assessment of Bariatric Surgery (Teen-LABS) study have demonstrated the effectiveness of MBS in adolescents [12]. The 2022 clinical practice consensus guidelines of the International Society for Pediatric and Adolescent Diabetes (ISPAD) recommended considering bariatric surgery as a therapeutic option for adolescents with obesity-related complications including T2DM, particularly in cases where drug therapy alone has proven ineffective [171]. Currently, two main indications for MBS are: firstly, BMI ≥ 32.5 kg/m^2^ or BMI ≥ 120% of P95, accompanied by severe obesity-related complications (such as moderate to severe obstructive sleep apnea syndrome, T2DM, PCOS, or severe fatty liver disease); secondly, BMI ≥ 37.5 kg/m^2^ with mild to moderate obesity-related complications. However, there is still a lack of data regarding reoperation rates following long-term metabolic surgery as well as bone health and nutritional deficiencies, and complications that may arise 10–15 years postsurgery [192]. Further follow-up and recording are necessary to address these gaps in knowledge.

At present, the primary applications of MBS in adolescents consist of vertical sleeve gastrectomy (VSG), Roux-en-Y gastric bypass (RYGB), pancreaticobiliary bypass surgery (BPD), and adjustable gastric band (AGB). BPD and AGB surgeries are infrequently performed due to a significantly elevated risk of reoperation compared to the other two. Nevertheless, some patients may experience postoperative complications after RYGB, including deficiencies in vitamins and trace elements. Compared to RYGB, VSG is a simpler option that is preferred by adolescents due to its lower risk of micronutrient deficiency and greater benefits for adolescents [193]. However, RYGB outperforms VSG in patients with severe reflux. Furthermore, in the experimental model of high-fat diet-induced obese mice, RYGB was found to modify obesity and insulin resistance by modulating early inflammatory response in the liver and WAT [194]. Overall, both VSG and RYGB are considered safe and effective therapeutic options for managing severe obesity in adolescents. According to the 2018 guidelines from the American Society for Metabolic and Bariatric Surgery (ASMBS), VSG has emerged as the most recommended surgery for adolescents [192].

Common short term complications following MBS include anastomotic leakage, increased gastroesophageal reflux, and intestinal obstruction. The most prevalent long term complications are nutritional deficiencies and postoperative psychological problems. A study on weight loss and health status among adolescents three years post-MBS revealed that the majority of participants experienced a reduction in their mean BMI by 25%–29% after surgery, with significant improvements observed in cardiovascular risk factors such as dyslipidemia, hypertension, and T2DM [195]. Therefore, for adolescents and their families, when considering surgical options, priority should be given to complications related to vitamin deficiency, persistence, and the need for reoperation. Finally, in accordance with the ASMBS 2018 guidelines, it is recommended that comprehensive and ongoing medical and behavioral support should be provided to all pediatric patients undergoing bariatric surgery, along with regular monitoring of trace elements, nutrition and metabolic status, mental health assessments, and psychological interventions [196].

### 6.4. Health Education

The development of insulin resistance is closely linked to environmental factors, thus, the variability of these factors presents an opportunity for preventing metabolic diseases [197]. Importantly, considering the limited adaptability of children to external fluctuations, the implementation of proactive and remedial measures should begin during gestation and continue throughout childhood, adolescence, and adulthood. For children, weight-loss programs should be individualized and lifestyle education should be adapted to local cultural norms [198]. Childhood should maintain a well-balanced diet, engage in appropriate physical activity, ensure sufficient sleep, undergo regular physical examinations, and be assessed for overweight or obesity to facilitate early detection of growth deviations and prompt intervention. The 2022 ADA standard recommends that children and adolescents who are overweight (BMI ≥ 85th percentile) or obese (BMI ≥ 95th percentile), and have one or more diabetes risk factors, should undergo risk-based screening for prediabetes or T2DM after the onset of puberty or at age 10 years [12]. Once hyperglycemia occurs, chronic complications may arise in all types of diabetic patients, albeit with varying rates of progression. Additionally, patients with prediabetes and diabetes are encouraged to receive individualized medical nutrition therapy to achieve treatment objectives, and frequent monitoring of blood glucose levels may be necessary for adolescents with T2DM [171]. In short, the child healthcare professionals should conduct a comprehensive assessment of the risk factors associated with childhood obesity and implement timely and effective interventions. Moreover, community physicians are responsible for conducting public education, delivering evidence-based treatment, promoting healthy lifestyles, and addressing the mental health needs of children and adolescents.

## 7. Conclusion

Obesity represents the foremost contributor to insulin resistance, which in turn exacerbates obesity, thereby creating a vicious cycle. The improvement of insulin resistance can be effectively achieved through weight loss, physical activity, and/or medication for hyperglycemia; however, it rarely returns to normal. Therefore, treatment for children with obesity is comprehensive and multifaceted, aiming to cultivate a healthy lifestyle that can effectively improve insulin resistance and enhance insulin sensitivity. In cases where patients have a poor response, pharmacological and metabolic surgical treatments may be considered to prevent chronic complications and premature mortality.

## Data Availability

The data/sources sourced for this literature review were acquired using publicly available PubMed (https://pubmed.ncbi.nlm.nih.gov) and Web of Science (https://www.webofscience.com/) databases.
